# Postoperative epidural hematoma as a rare complication after intracranial tumor resection: a case series report and causes analysis

**DOI:** 10.1186/s41016-024-00359-2

**Published:** 2024-03-01

**Authors:** Minghui Zeng, Zhijin Li, Chunsheng Xia, Xufeng Cheng, Yehan Wang, Fei Wang

**Affiliations:** Department of Neurosurgery, The First Affiliated Hospital of USTC (Anhui Provincial Hospital), Division of Life Science and Medicine, University of Science and Technology of China, Hefei, 230036 Anhui China

**Keywords:** Epidural hematoma, Postoperative complication, Craniotomy, Brain tumor

## Abstract

**Background:**

To review the treatment and the causes of postoperative epidural hematoma (PEDH) after intracranial tumor resection.

**Method:**

A retrospective case study was conducted to examine a series of patients who developed PEDH as a complication following intracranial tumor resection between January 2016 and June 2021. The study collected data from hospital charts, including clinical status at admission, imaging results, histopathologic findings, surgical management, complications, and outcomes. Causes of PEDH were evaluated through a review of operative notes and discussions with the surgical team.

**Results:**

Twenty-five patients (10 males, 15 females; median age 42 years, range 11–61 years; median medical history 27 months, range 1–96 months) were enrolled in the study. Regarding tumor location, 16 cases exhibited supratentorial brain tumors, 4 cases had infratentorial brain tumors, 2 cases of tumors occurred in the petroclival region, 2 cases in the peritorcular region, and 1 case in the pineal region. Four of these cases were complicated with supratentorial hydrocephalus. The 25 cases in this study were classified into four types based on location. Type 1 refers to EDHs that occur at the adjacent site of the operative field without involvement of the surgical area. Type 2 includes hematomas that occur at the adjacent site of the surgical area and the surgical area. Type 3 includes EDHs that occur in distant areas, and type 4 involves EDHs in the surgical field. The numbers of cases of types 1, 2, 3, and 4 PEDHs were 16, 2, 3, and 4 cases, respectively. Most PEDHs were associated with reduced ICP after craniotomy due to intracranial tumor resection and substantial loss of CSF. All patients achieved satisfactory outcomes after hematoma evacuation.

**Conclusion:**

The decrease in ICP resulting from intracranial tumor resection and CSF loss might lead to PEDHs. By employing optimized surgical techniques and meticulous patient management to prevent rapid decreases in ICP and dural detachment, we can potentially lower the incidence of PEDHs. Additionally, prompt evacuation of hematomas can contribute to positive outcomes.

## Background

The postoperative epidural hematomas (PEDHs) that have garnered recent attention are those large enough to compress brain parenchyma and cause neurological symptoms, necessitating secondary surgery for hematoma evacuation. PEDH occurs as a serious, sometimes lethal, complication that can be found in approximately 1–2% of intracranial tumor resection [1–3]. Fukamachi et al. [3] conducted a review of 16 cases of PEDHs, 10 of which underwent hematoma evacuation, and determined that aggressive hematoma evacuation can result in positive patient outcomes. Jinlu Yu [2] examined 14 cases of remote epidural hematoma as a postoperative complication following intracranial tumor resection, with all patients undergoing hematoma evacuation. Thirteen patients made a good recovery and were discharged, while one patient died. PEDH typically occurs acutely, preceding herniation. Early detection of PEDH can facilitate earlier intervention, necessitating awareness of the risk of PEDH. In this article, we present a review of 25 cases of PEDHs and analyze their causes.

## Methods

### Patients

This retrospective case series includes patients treated in the Department of Neurosurgery at the First Affiliated Hospital of the University of Science and Technology of China between January 2016 and June 2021. The patients developed PEDHs as a complication of non-traumatic craniotomy. The inclusion criteria were as follows: (1) patients underwent craniotomy for the treatment of brain tumors; (2) patients required additional surgery due to the development of PEDH as a complication. The data were collected from the patients’ medical records, including information on their clinical status at admission, imaging results, histopathological findings, surgical management, complications, and outcomes. Causes of PEDH were evaluated through a review of operative notes and discussions with the surgical team. Preoperative assessments consisted of computerized tomography (CT), magnetic resonance imaging (MRI), and/or CT angiography (CTA). As this was a retrospective case series, only patients who met all the inclusion criteria were included in the analysis. Given the retrospective nature of this study, the ethics committee of our hospital approved it as exempt from requiring informed consent from the patients.

### Treatment

#### Tumor resection

The surgical approach was carefully selected based on the location of the tumor. Following tumor resection, warm saline was administered into the surgical cavity during the suturing of the dura, ensuring the elimination of any trapped air. Holes were drilled around the edge of bone window for a stay suture of the dura and hemostasis. A closed epidural drainage tube was set routinely. Postoperatively, cerebrospinal fluid (CSF) drainage was carefully regulated to avoid excessive drainage, especially in cases involving ventricular drainage.

There are more than 80 neurosurgeons in our center, and different doctors may have slight differences in surgical methods.

#### Epidural hematoma evacuation

Immediately after the craniotomy surgery and 48 hours later, CT examinations were carried out. If the PEDH was large enough to cause compression of the brain parenchyma and neurological symptoms, a craniotomy for hematoma evacuation was conducted. Following the evacuation of the EDH, the dura was sutured in place with precise hemostasis, and a routine set of postoperative epidural drainage tubes was inserted.

#### Postoperative treatment and follow-up

After the surgery, patients received conventional treatment, which was identical to that given to patients without PEDHs. Consciousness levels and any changes in physical activity were closely observed. Routine head CT scans were conducted. Once patients had recovered, they were discharged and followed up using the Karnofsky Performance Status (KPS) scoring system through two telephone interviews.

### Statistical analysis

All data were analyzed using SPSS 19.0 (SPSS Inc., Chicago, IL, USA). The data were presented as mean ± SD.

## Results

### Clinical baseline data

Twenty-five patients (ten males, fifteen females; average age 38.3 ± 15.8 years, range 11–61 years; medical history 1–96 months) were enrolled (Table 1). Three patients had a prior history of tumor resection. The preoperative Karnofsky Performance Status (KPS) scores were 90 for 23 patients and 80 for 2 patients. The tumor locations were diverse, with 16 cases of supratentorial brain tumors, 4 cases of infratentorial brain tumors, 2 cases in the petroclival region, 2 in the peritorcular region, and 1 in the pineal region. Four cases were complicated with supratentorial hydrocephalus. The tumor types included 11 meningiomas, 7 gliomas, 2 cavernous angiomas, 2 craniopharyngiomas, 1 acoustic schwannoma, 1 choroid plexus papilloma, and 1 metastatic adenocarcinoma. Eight cases (16%) had a tumor diameter exceeding 70 mm.

**Table 1:** Summary of clinical baseline data

Characteristic	Details	
Total patients (*N*)	25	
Gender	Male	10
	Female	15
Average age (year)	38.3 ± 15.8	
Medical history (months)	Median 11 (range 1–96)	
Preoperative KPS score (*N*)	90	23
	80	2
Tumor location (*N*)	Supratentorial	16
	Infratentorial	4
	Petroclival	2
	Peritorcular	2
	Pineal	1
Tumor types (*N*)	Meningioma	11
	Glioma	7
	Cavernous angioma	2
	Craniopharyngioma	2
	Acoustic schwannoma	1
	Choroid plexus papilloma	1
	Metastatic adenocarcinoma	1
Tumor diameter (mm)	49.4 ± 24.5	
Bone flap area (cm^2^)	54.9 ± 29.4	
Operation time (minutes)	405.6 ± 168.5 (range 140–738)	
Blood loss (ml)	492 ± 464 (range 50–2000)	
Volume of PEDHs (ml)	65.5 ± 37.8	

**Fig. 1:**
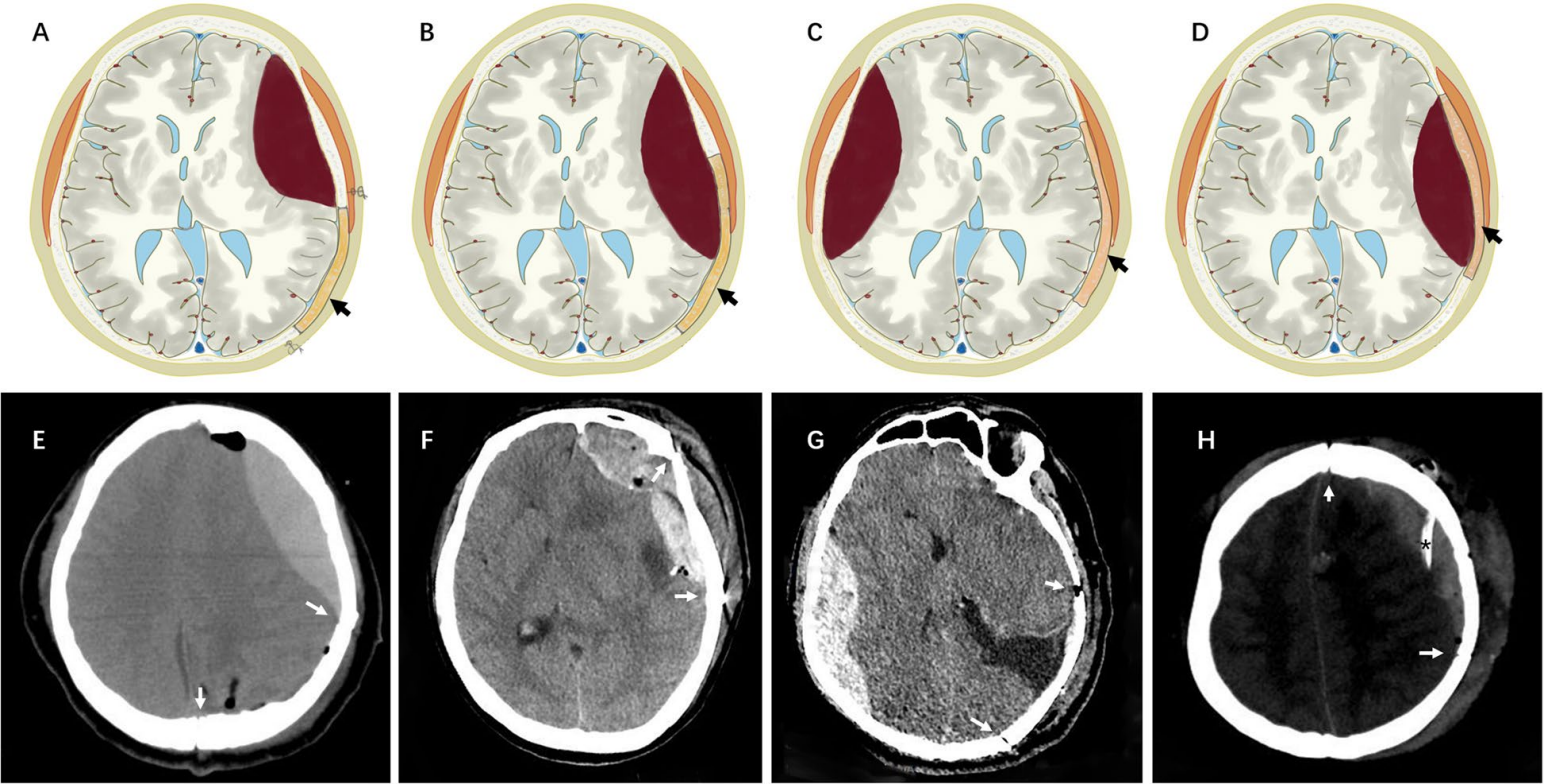
Schematic representation of the four types of PEDH based on the location. Type 1 PEDH: hematoma formation adjacent to the surgical site, without involvement of the surgical area (**A**, **E**). Type 2 PEDH: hematoma at both the adjacent site and within the surgical area (**B**, **F**). Type 3 PEDH: EDH occurring in remote areas or on the contralateral side of the surgical area (**C**, **G**). Type 4 PEDH: hematoma within the surgical field (**D**, **H**). The black arrows in **A**, **B**, **C**, and **D** indicate bone flaps. The white arrows in **E**, **F**, **G**, and **H** demarcate bone flaps. The asterisk in **H** highlights the epidural drainage tube

After analyzing 25 cases, we found that these PEDHs could be classified based on their location. The first type includes EDHs that occur at the adjacent site of the operative field without the involvement of the surgical area. The second type includes hematomas that occur both at the adjacent site and within the surgical area. The third type involves EDHs that occur in distant areas, and the fourth type involves EDHs within the operative field (Fig. 1). The distribution of these types among the cases is as follows: type 1 PEDH in 16 cases, type 2 in 2 cases, type 3 in 3 cases, and type 4 in 4 cases.

The causes of hematoma formation, as documented in the surgical records and as reported by surgeons during interviews, are presented in Table 2. Among the 16 patients with type 1 PEDHs, the intracranial pressure (ICP) significantly decreased due to the rapid aspiration of CSF from the ventricle, lumbar drainage, or the basal cistern, as well as fluid from cystic tumors in 10 patients. Six patients had tumors larger than 7 cm in diameter. Three cases were complicated by supratentorial hydrocephalus. The bleeding sources documented in the operative notes included dural oozing in 9 patients, while the source remained unknown in 7 patients.

**Table 2:** Sources of bleeding of PEDHs in surgical records, and causes of bleeding as interviewed with surgeons

PEDHs type (*N*)	Source of bleeding in surgical record (*N*)	Cause of bleeding as interviewed with surgeon (*N*)
Type 1 (16)	Dural oozing (9) Unknown (7)	Giant tumor size (6) CSF release (10) Hydrocephalus (3) Massive blood loss (1)
Type 2 (2)	Occipital muscle oozing (1) Dural bleeding adjacent to the sagittal sinus (1)	Stay sutures failure (2) Incomplete hemostasis of muscle artery (1)
Type 3 (3)	Dural oozing (2) Unknown (1)	Giant tumor size (2) CSF release (3)
Type 4 (4)	Dural oozing (1) Occipital muscle oozing (1) Unknown (2)	Underperformance of central stay sutures (4) Postoperative ICP reduced via lumbar drainage (2) Inappropriate use of diuretics (1)

Giant tumor size: tumor with a diameter of 70 mm or greater

Two patients with type 2 PEDHs experienced the failure of stay sutures. The bleeding sources documented in the operative notes included occipital muscle oozing in 1 patient and bleeding in the sagittal sinus in another patient.

The 3 patients with type 3 PEDHs experienced brain collapse due to the rapid aspiration of CSF from the ventricle or the basal cistern. Two of these patients had tumors larger than 7 cm in diameter. The bleeding sources documented in the operative notes included dural oozing in 2 patients, while the source remained unknown in 1 patient.

In the 4 patients with type 4 PEDHs, the ICP was rapidly reduced due to aspiration of CSF from the ventricle, lumbar drainage, or the basal cistern. When the bone flaps were closed, the central stay sutures of the four patients were not tied. Lumbar drainage was performed in 2 patients due to suspected intracranial infection 48 hours postoperatively. The bleeding sources documented in the operative notes included dural oozing in one patient, oozing from the occipital muscle in another patient, and 2 patients had unknown sources.

### Surgery outcomes

Nineteen out of 25 patients had gross total resection of the tumors. The average surgical time for tumor resection was 405.6 ± 168.5 min (range 140–738 min) and the average blood loss was 492 ± 464 mL (range 50–2000 mL).

### Treatment results

**Fig. 2:**
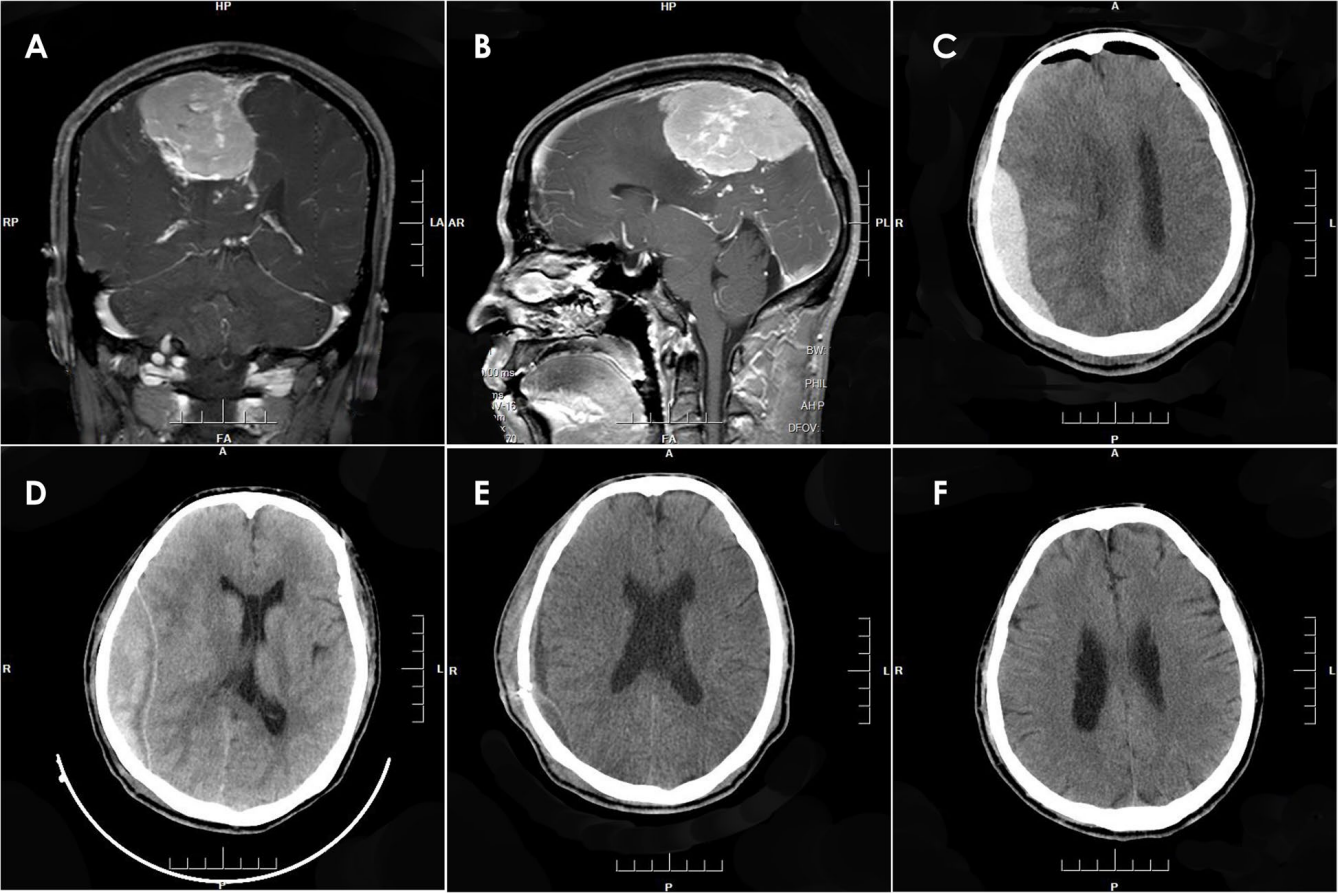
MRI with contrast enhancement revealing a giant parasagittal meningioma (**A** and **B**). CT scan showing a type 1 PEDH on the left frontal-parietal lobe (**C**). CT scan demonstrating the liquefaction of the EDH 2 weeks later (**D**). After the EDH evacuation via burr hole and drainage procedure, a CT scan was obtained (**E**). CT scan obtained 3 months later (**F**)

**Fig. 3:**
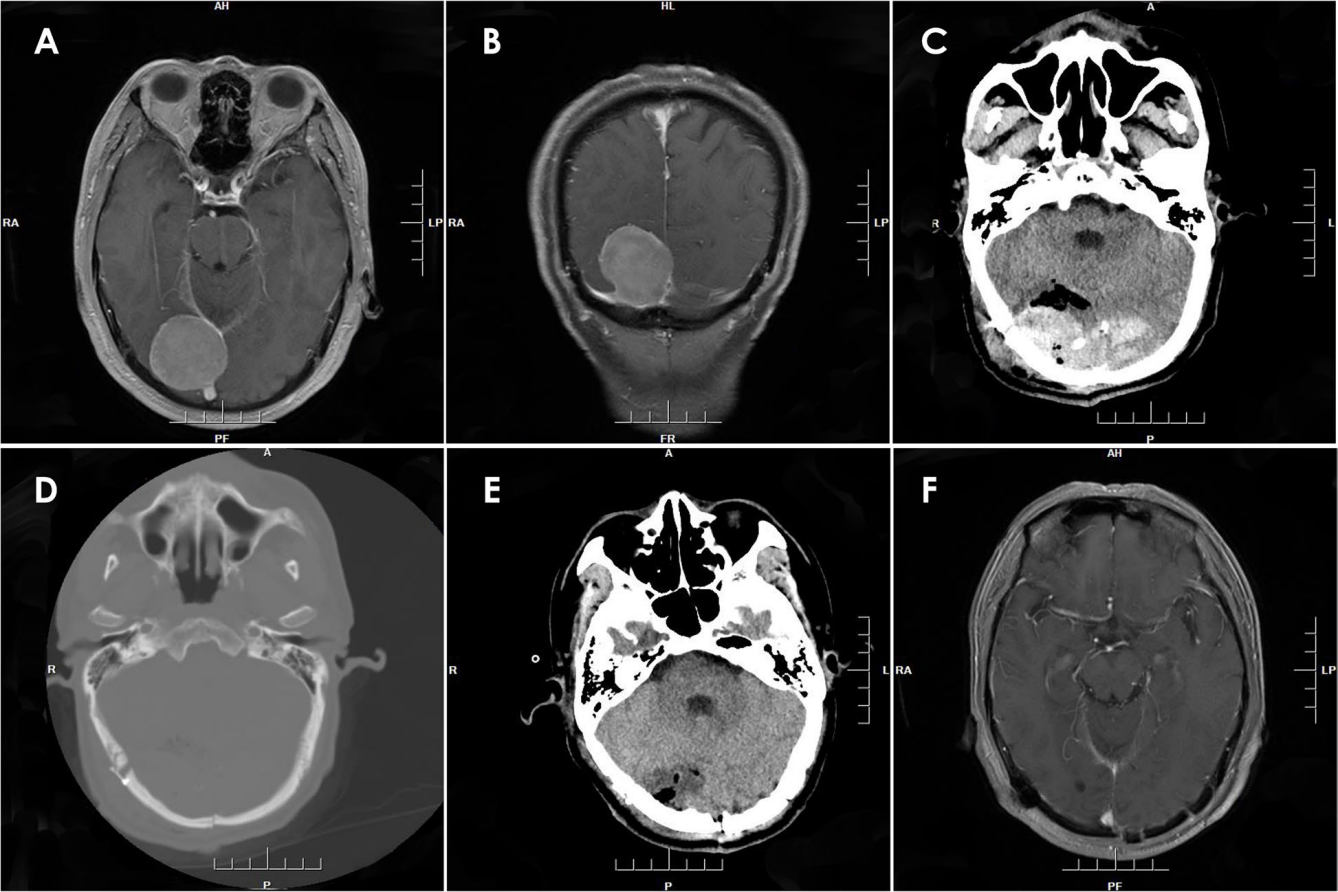
MRI with contrast enhancement revealing a right peritorcular meningioma (**A** and **B**). A type 2 PEDH on the bilateral occipital lobe (**C**). The causes of PEDH were attributed to the failure of dura suspension and bleeding in the sagittal sinus, according to the operative note. The CT bone window showed that the bone flap reached the midline, possibly leading to incomplete suspension due to the sagittal sinus (**D**). The EDH was evacuated (**E**). MRI with contrast enhancement obtained 3 months after surgery (**F**)

**Fig. 4:**
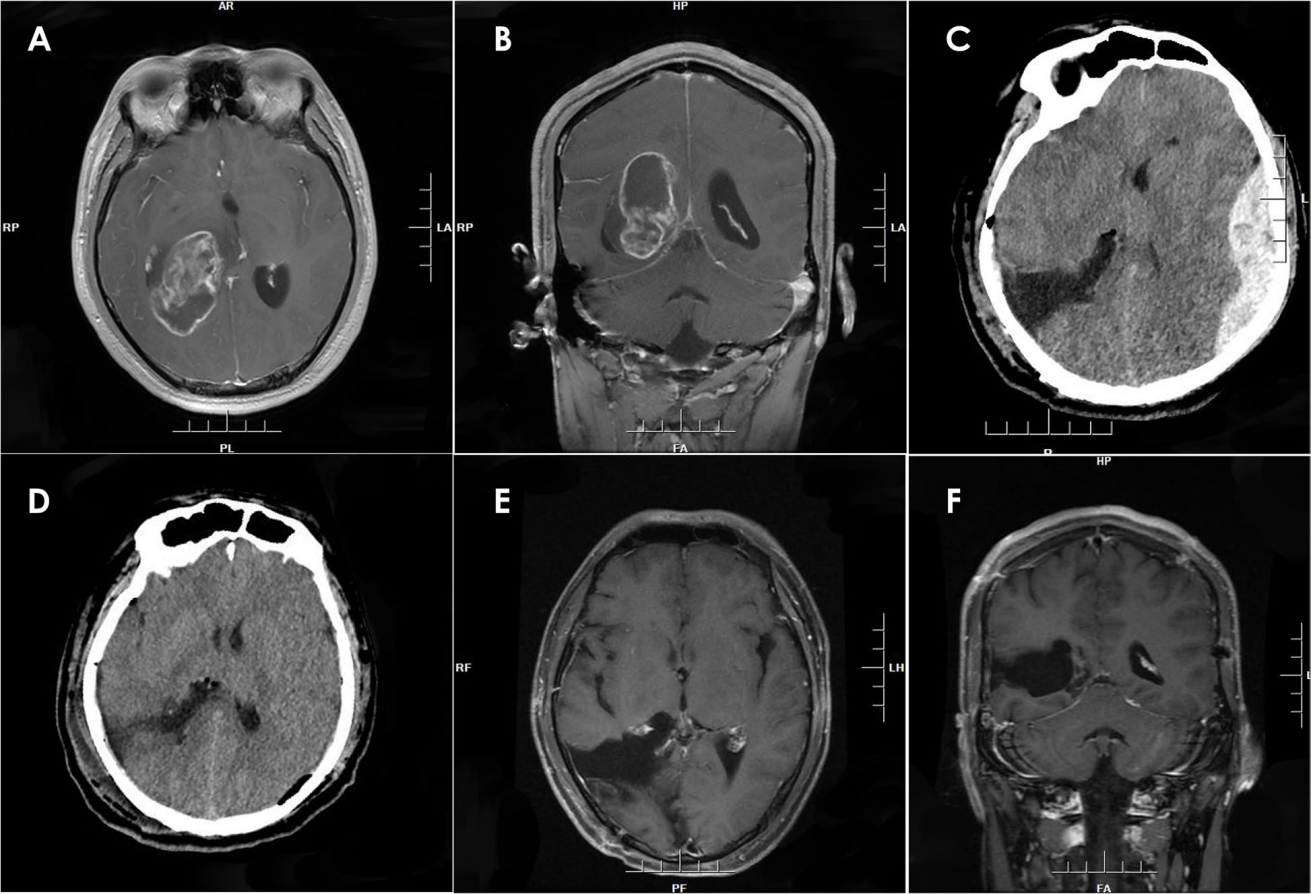
The enhanced MRI showed a tumor in the right ventricle (**A**, **B**). The postoperative CT showed PEDH on the contralateral (left) temporal-parietal-occipital region (**C**). CT of the patient following acute extradural hematoma evacuation surgery (**D**). And enhanced MRI (**E**, **F**) 3 months post-surgery was obtained for a follow-up examination

**Fig. 5:**
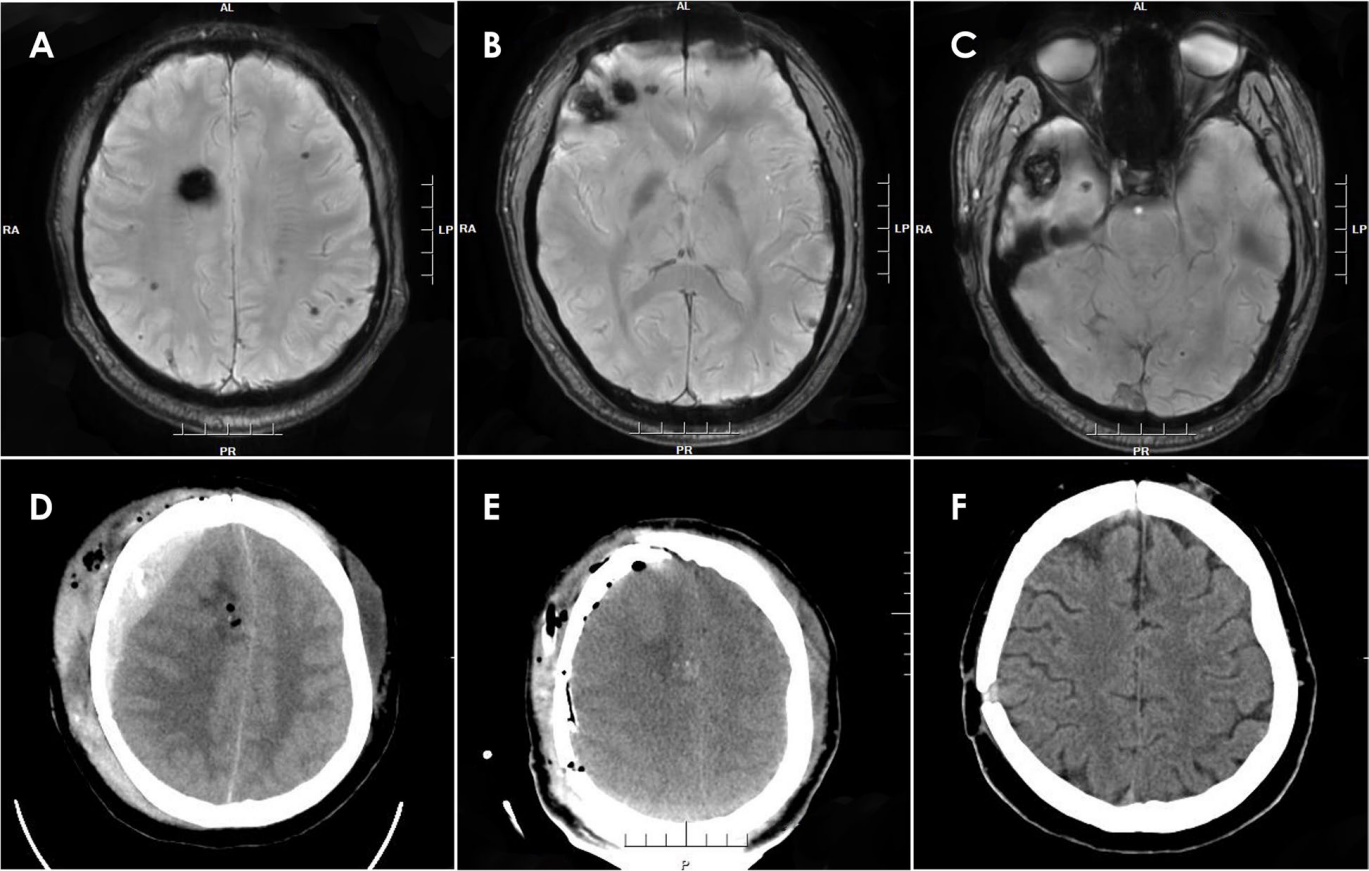
SWI revealing multiple cavernous angiomas (**A**, **B**, and **C**). CT scan demonstrating a type 4 PEDH-epidural hematoma located under the surgical field following tumor resection (**D**). CT scan showing that the hematoma had been successfully evacuated (**E**). CT scan obtained 3 months postoperatively (**F**)

Craniotomies were performed in 24 cases of PEDHs for hematoma evacuation, while one patient received a burr hole for drainage (Fig. 2, Case 16). All 25 patients who underwent both intracranial tumor resection and EDH evacuation recovered well and were discharged. Postoperative KPS scores at 3 months were 90 in 22 cases and 80 in 3 cases. The typical cases of PEDH are depicted in Figs. 2, 3, 4, and 5.

## Discussion

PEDH, as a rare and serious postoperative complication after intracranial tumor resection, may worsen the prognosis of patients [2, 4]. Therefore, more awareness is required regarding the risk of PEDH. In this article, we present a comprehensive review of 25 cases of PEDH, accompanied by an analysis of the underlying causes. We have observed a significant association between PEDH and the reduction of ICP following craniotomy due to intracranial tumor resection and substantial loss of CSF in most cases. In this study, we focused on the impact of surgical procedures on the development of PEDHs. On the one hand, the postoperative hematoma is suggested to be an avoidable complication of intracranial surgery [5]. On the other hand, we recognize that surgical procedures cannot always be performed with complete uniformity, and there are numerous individual differences and unforeseen circumstances that arise during the operation. Examining the surgeon’s interviews and surgical records for insights into the causes of epidural hematoma may provide valuable subjective and empirical data that can enhance the surgical skills of young doctors.

**Fig. 6:**
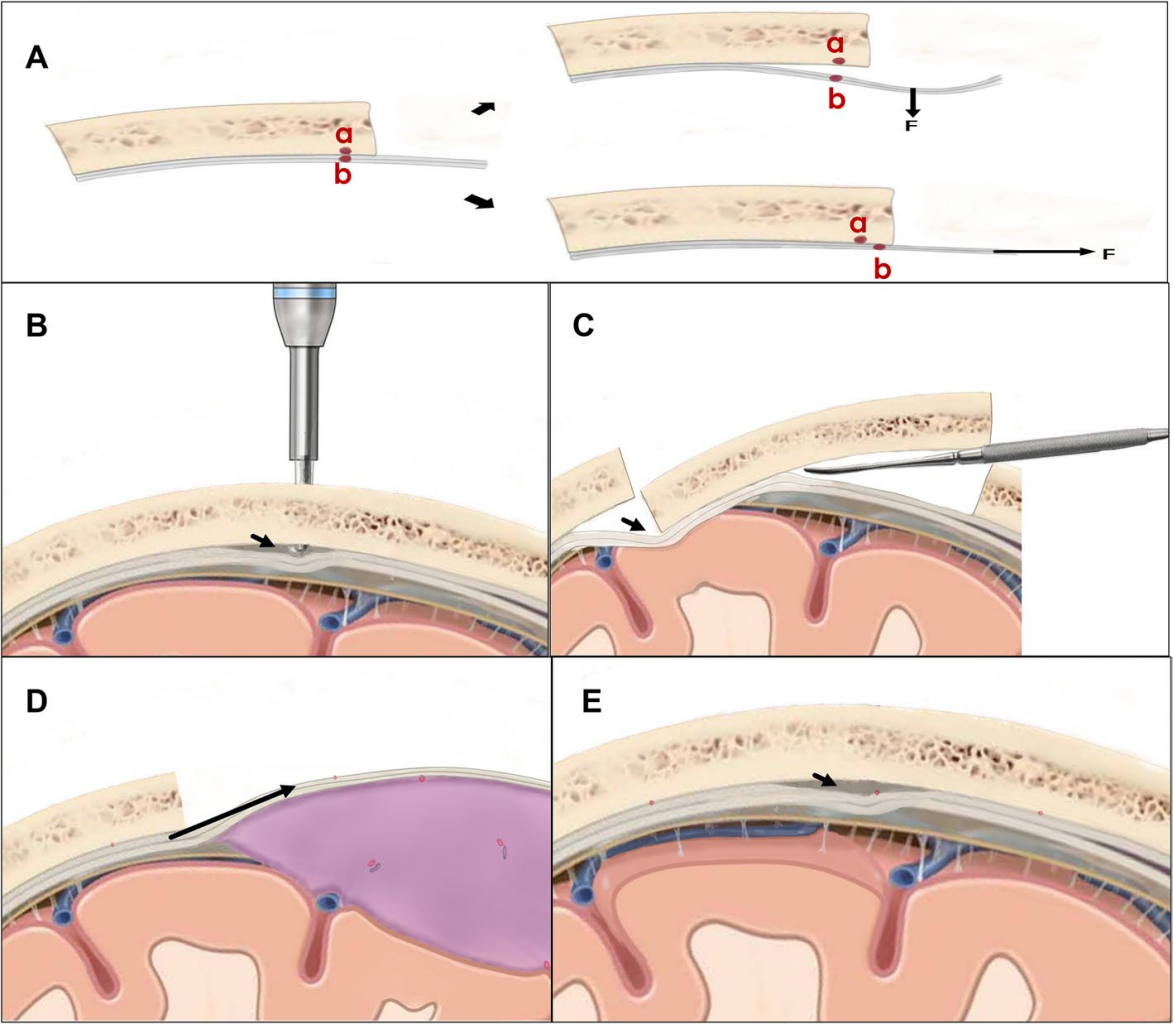
Different reasons lead to dural stripping. There are two distinct methods of separating the dura from the inner table of the skull. The relative displacement between the inner skull plate (red dot a) and the dura (red dot b) may result in dural detachment, which can cause extradural vessel hemorrhage. The vertical and parallel direction relative displacement is caused by forces in different directions (**A**). The use of a milling cutter can cause dural stripping (**B**). Downward pressure is produced by the lever principle when using the Penfield dissector to remove the bone flap (**C**). When exposing a giant meningioma, due to the large bone window and intracranial hypertension, intracranial content bulges through the opening, leading to the displacement of the dura and bone window margin in a parallel direction (**D**). Falling intracranial pressure causes the cortex to collapse and result in dural detachment (**E**)

**Fig. 7:**
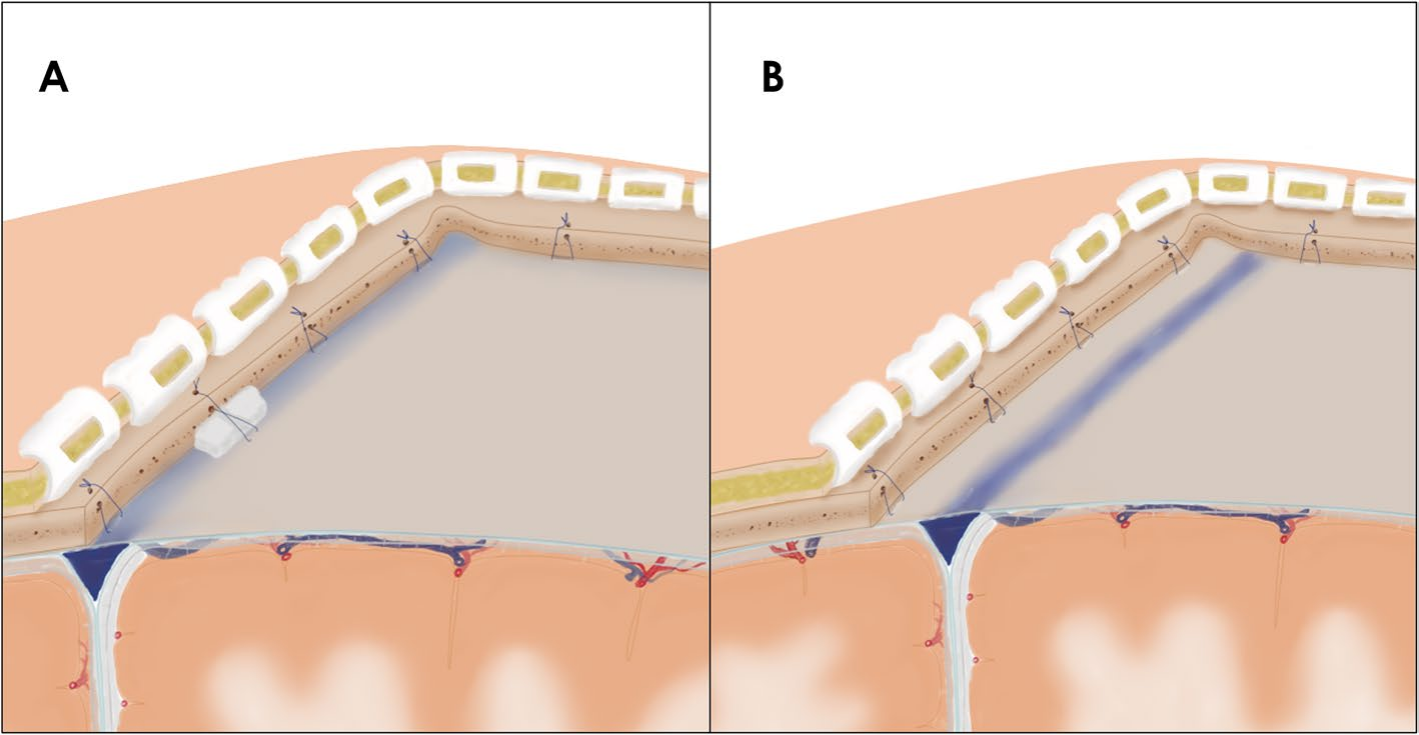
It can be difficult to place close subdural stay sutures when the dural suspension involves the sagittal sinus (**A**). Fully exposing the sagittal sinus makes it easier to place stay sutures (**B**)

Dura mater-bone separation is considered a fundamental step in the development of extradural hematomas [6]. The separation of the surrounding adjacent dura from the inner skull plate during surgery (Fig. 6) acts as the most critical initiating factor of PEDH. Additionally, the surgical region is typically situated at the highest point of the brain, resulting in significant transmural pressure on the dura adjacent to the surgical area, along with significant stretching of the bridging veins [2]. Consequently, PEDHs are more likely to occur on the ipsilateral side of the surgical region. Dural suspension serves as a primary measure to prevent the development of EDHs, which can occur due to the absence of a close subdural stay suture [7]. For type 1 PEDH, which occurs on a site adjacent to the surgical region, the stay suture of the dura surrounding the surgery area prevents the spread of the hematoma toward the surgical area and the subsequent extension to distant areas [2]. Type 2 PEDH is most likely to occur with an incomplete dural suspension, particularly when it involves the sagittal sinus. Fully exposing the sagittal sinus before suspension can be a beneficial approach (Fig. 7).

In several published studies the etiology of PEDH has been discussed. It is widely accepted that intracranial tumor resection and the substantial loss of CSF lead to a decrease in ICP. This reduction in ICP results in an increase in dural venous transmural pressure and disrupts vascular regulation, ultimately causing blood vessel rupture [4, 8]. The pressure effect produced by the hematoma increases the transmural venous pressure, aggravating the bleeding and resulting in hematoma expansion [9]. In our case series, type 3 PEDHs were predominantly associated with decreasing ICP resulting from CSF loss. Other case reports have described EDHs following ventriculoperitoneal shunt (VPS) [10], lumbar puncture [11], and surgical removal of a lumbosacral schwannoma [12]. The development of PEDHs can be attributed to ICP drops and brain shifts related to decompression, resulting in a reduction of the tamponade effect on torn dural arteries or veins [13, 14]. Odake et al. [15] discussed 43 supratentorial PEDH cases after CSF drainage for ventricular decompression and reported a higher risk in young and middle-aged patients, aged 10 to 40 years [15]. The average age of the patients in this study was 38.3 years old, while the average age of patients with type 3 PEDH was 24.7 years. Theoretically, the dura mater in young patients may detach more easily. What is more, type 3 PEDH can also be caused by the use of a skull clamp [16, 17].

The development of 4 cases of type 4 PEDHs was primarily attributed to incomplete hemostasis of the dura mater or muscle in 2 cases and decreased ICP due to continuous lumbar drainage in the other 2 cases. In all four cases, the non-performance of central stay sutures might be another contributing factor. One of the most widely accepted hypotheses regarding the development of EDH involves the tamponading effect initially provided by increased ICP on vessels involved in detached dura. The loss of this tamponade effect, resulting from a reduction in ICP due to resection of intracranial tumors, edema treatment with hyperventilation and/or hyperosmotic diuretics, decompressive craniectomy, or CSF drainage, has been proposed as a determining factor in the development of EDH [18]. To prevent type 4 PEDHs, it is essential to achieve complete hemostasis of the muscle, dura mater, and bone. Central stay sutures should be performed, and the patency of the extradural drain should be regularly monitored [3]. Excessive drainage should be avoided when introducing lumbar drainage.

The presence of a large intra-operative blood loss (exceeding 800 ml) and a wide craniotomy area (equal to or greater than 71.53 cm^2^) have been strongly associated with the development of PEDH [1]. It is hypothesized that a significant blood loss during surgery may result in the development of coagulopathy, such as hemodilution, loss of coagulation factors, platelet dysfunction, or other issues that can lead to massive transfusion complications [19]. In this case series, approximately 32% (*N* = 8) of cases had a wide craniotomy area (equal to or greater than 71.53 cm^2^), and 16% (*N* = 4) of patients experienced blood loss exceeding 800ml. The presence of a wide craniotomy area and significant blood loss during surgery might also be linked to large-sized tumors, as dural detachment is more likely during craniotomy for such tumors (Fig. 6D). Furthermore, the reduction in ICP following resection of a large tumor can lessen the tamponade effect on torn dural vessels, leading to the development of PEDH.

In cases of acute EDH, symptoms can arise rapidly; however, patients may remain asymptomatic with slow bleeding. In this study, type 4 PEDH developed gradually, and the decision to evacuate the hematoma was made 12 h, 2 days, 4 days, and 9 days after the previous surgery, respectively. One patient with mild symptoms (mild decrease in strength in the left limbs) received a burl hole for drainage 2 weeks later and made a good recovery (Fig. 2). Zhao X [20] reported 33 cases of EDH treated by CT-guided brain puncture, considering it a simple, fast, and accurate positioning method. For certain patients, minimally invasive surgery could be considered as an alternative for the treatment of PEDHs.

## Limitation

The current study’s limitations are primarily due to the small sample size, the absence of a control group, and inherent limitations that exist in retrospective case studies. In addition, this study obtained the causes of PEDH through surgical records and interviews with surgical physicians, focusing on the role of operators in the occurrence and prevention of PEDH. However, due to the lack of statistical evidence, this may be subjective.

## Conclusions

The decrease in ICP resulting from intracranial tumor resection and CSF loss might lead to PEDHs. By employing optimized surgical techniques and meticulous patient management to prevent rapid decreases in ICP and dural detachment, we can potentially lower the incidence of PEDHs. Additionally, prompt evacuation of hematomas can contribute to positive outcomes.

## Data Availability

The data that support the findings of this study are available from the corresponding author upon reasonable request.
